# Novel strategies targeting mitochondria-lysosome contact sites for the treatment of neurological diseases

**DOI:** 10.3389/fnmol.2024.1527013

**Published:** 2025-01-14

**Authors:** Yinyin Xie, Wenlin Sun, Aoya Han, Xinru Zhou, Shijie Zhang, Changchang Shen, Yi Xie, Cui Wang, Nanchang Xie

**Affiliations:** ^1^Department of Neurology, The First Affiliated Hospital of Zhengzhou University, Zhengzhou, China; ^2^Department of Clinical Laboratory, The First Affiliated Hospital of Zhengzhou University, Key Clinical Laboratory of Henan Province, Zhengzhou, China

**Keywords:** lysosomal dynamics, mitochondria-lysosome contact sites, mitochondrial network, mitophagy, neurological diseases, substance exchanges

## Abstract

Mitochondria and lysosomes are critical for neuronal homeostasis, as highlighted by their dysfunction in various neurological diseases. Recent studies have identified dynamic membrane contact sites between mitochondria and lysosomes, independent of mitophagy and the lysosomal degradation of mitochondrial-derived vesicles (MDVs), allowing bidirectional crosstalk between these cell compartments, the dynamic regulation of organelle networks, and substance exchanges. Emerging evidence suggests that abnormalities in mitochondria-lysosome contact sites (MLCSs) contribute to neurological diseases, including Parkinson’s disease, Charcot–Marie-Tooth (CMT) disease, lysosomal storage diseases, and epilepsy. This article reviews recent research advances regarding the tethering processes, regulation, and function of MLCSs and their role in neurological diseases.

## 1 Introduction

The idea that mitochondria are merely “cellular energy factories” has now been firmly abandoned. Extensive research has shown that mitochondria play crucial roles in the regulation of calcium homeostasis, apoptosis, cell growth, differentiation, and reactive oxygen species (ROS) production (Claiborne et al., 2016; Harrington et al., 2023; Markovinovic et al., 2022). Over the past decade, the crosstalk between mitochondria and other organelles has garnered widespread attention (Valm et al., 2017), with considerable progress being made in the study of mitochondria-lysosome contact sites (MLCSs). Although contact sites between mitochondria and vacuoles had been discovered in yeast as early as 2014 (Elbaz-Alon et al., 2014), it was not until 2018 that Wong et al. using high-resolution microscopy, first identified in mammalian cells the direct physical contact between mitochondria and lysosomes, known as MLCSs (Wong et al., 2018).

**Figure 1:**
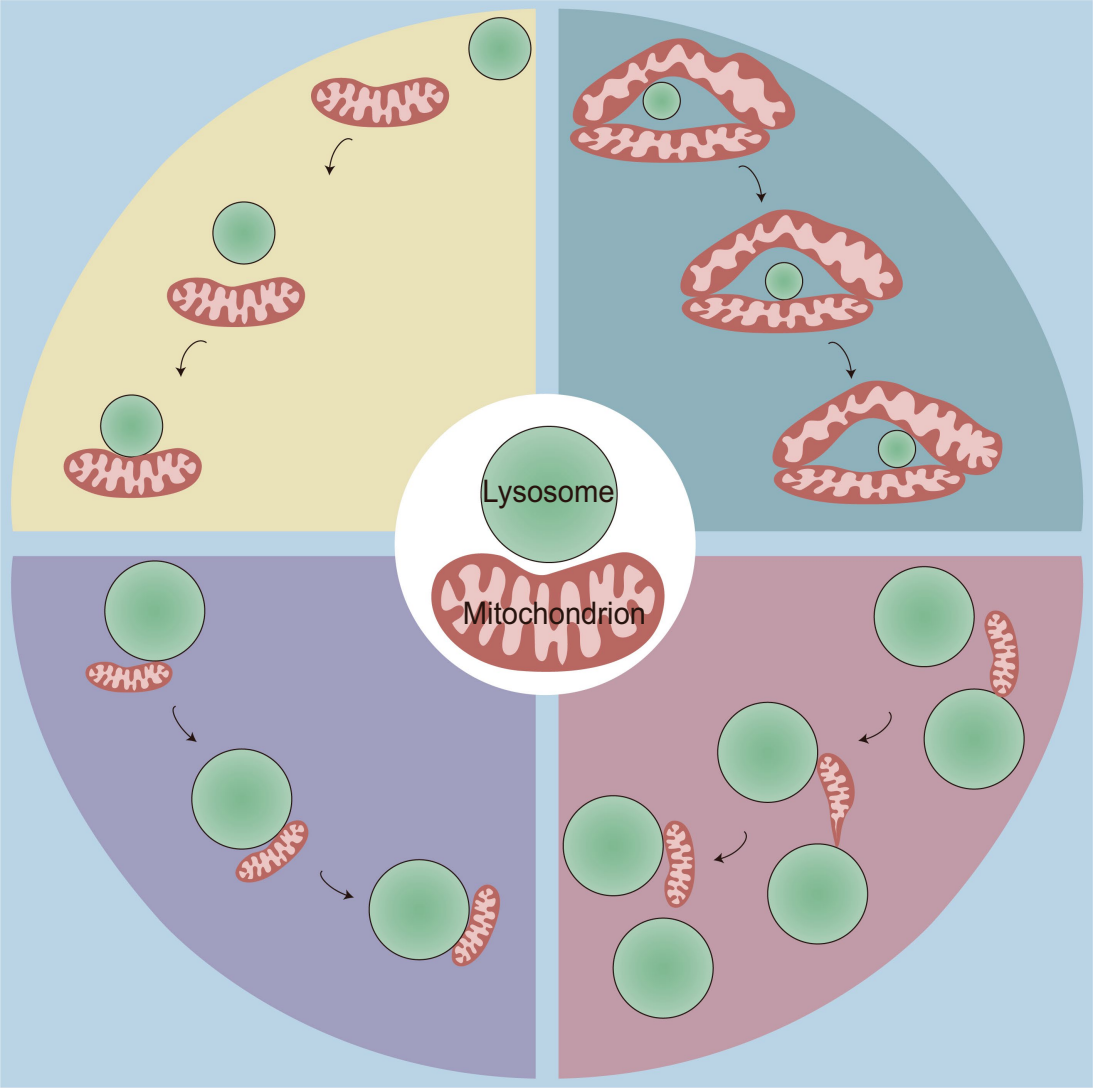
Four types of physical mitochondria-lysosome interactions. This figure illustrates four distinct types of dynamic contact between mitochondria and lysosomes, highlighting the spatial and temporal dynamics of MLCS, which are crucial for cellular homeostasis and organelle function. Top Left: rapid lysosomal movement followed by adhesion: some lysosomes initially exhibit rapid movement before contacting with and adhering to mitochondria; Top Right: motility-restricted interaction: the mitochondrial network surrounds certain types of lysosomes, limiting their movement along it; Bottom Left: lysosome-bound mitochondrial transport: smaller mitochondria attach to lysosomes and move together as a unit within the cytoplasm; Bottom Right: mitochondrial transfer between lysosomes: this interaction involves the dynamic transfer of a mitochondrion between two lysosomes. Initially, one end of the mitochondrion adheres to a lysosome, and the mitochondrion begins to elongate. It then detaches from the first lysosome and transfers to the second lysosome, forming a thin and tubular intermediate structure during the transfer.

Consistent with the range of 10–30 nm at other contact sites (Phillips and Voeltz, 2016; Wu et al., 2018), MLCSs exhibit an average intermembrane distance of ~10 nm (Kim et al., 2021; Wong et al., 2018). Depending on the cell type, these contacts have variable tethering durations, with stable tethering lasting on average 60 s (Wong et al., 2018), but extending up to 13 min (Han et al., 2017). MLCSs represent a novel form of interorganellar crosstalk that is distinct from mitophagy and lysosomal degradation of mitochondrial-derived vesicles (MDVs). These contact sites do not express key autophagosome markers (ULK1, ATG5, ATG12, and LC3) (Wong et al., 2018) and are not disrupted by knockout of autophagy receptors (NDP52, OPTN, NBR1, TAX1BP1, and p62) (Chen et al., 2018). Moreover, no substantial transfer of mitochondrial proteins to lysosomes at these contact sites has been observed, and mitochondria that form these contacts, which contain both outer mitochondrial membrane (OMM) and matrix proteins (Wong et al., 2018), are larger than MDVs (Wong et al., 2019a). The development of cell-permeable organic fluorescent probes for live-cell long-term super-resolution imaging enabled the identification of the following four types of physical mitochondria-lysosome interactions: (1) rapid lysosomal movement followed by adhesion; (2) motility-restricted interaction; (3) lysosome-bound mitochondrial transport; and (4) mitochondrial transfer between lysosomes (Figure 1; Han et al., 2017). Regarding the contact distance, at least two types of MLCSs are known: narrow/short-range interactions (~4 nm) and wide/long-range interactions (~10 nm) (Giamogante et al., 2024). These interactions exhibit distinct responses to mitophagy or autophagy stimuli and to different levels of specific tethering or untethering factors (Giamogante et al., 2024). Furthermore, a decrease in extracellular matrix stiffness leads to rearrangement of the cellular cytoskeleton, which may facilitate random movements of lysosomes along the disordered cytoskeleton, thereby increasing the probability of MLCSs (Wang K. et al., 2024). Although these observations demonstrate the dynamic and varied nature of mitochondria-lysosome interactions, their underlying molecular mechanisms and physiological significance remain unclear. Future research is needed to elucidate the specific signaling pathways, regulatory proteins, and metabolic processes that govern these interactions under defined conditions and to explore their broader implications for cellular function and homeostasis.

Although the mitochondrial and lysosomal membranes form specific contact sites mediated by tethering proteins, it is important to emphasize that they do not fuse, but instead maintain their distinct structures and characteristics, which are crucial for their roles as major platforms in regulating various physiological processes, such as the mitochondrial network, lysosomal dynamics, calcium homeostasis, and lipid metabolism, all of which have been implicated in Parkinson’s disease (PD), Charcot–Marie-Tooth (CMT) disease, and lysosomal storage diseases (LSDs) (Cantarero et al., 2021; Cisneros et al., 2022; Giamogante et al., 2024; Peng et al., 2020; Pijuan et al., 2022). This review focuses on recent evidence regarding MLCS tethers, regulation, and functions, as well as their potential involvement in various neurological diseases.

## 2 Tethering and regulation of mitochondria-lysosome contact sites

**Figure 2:**
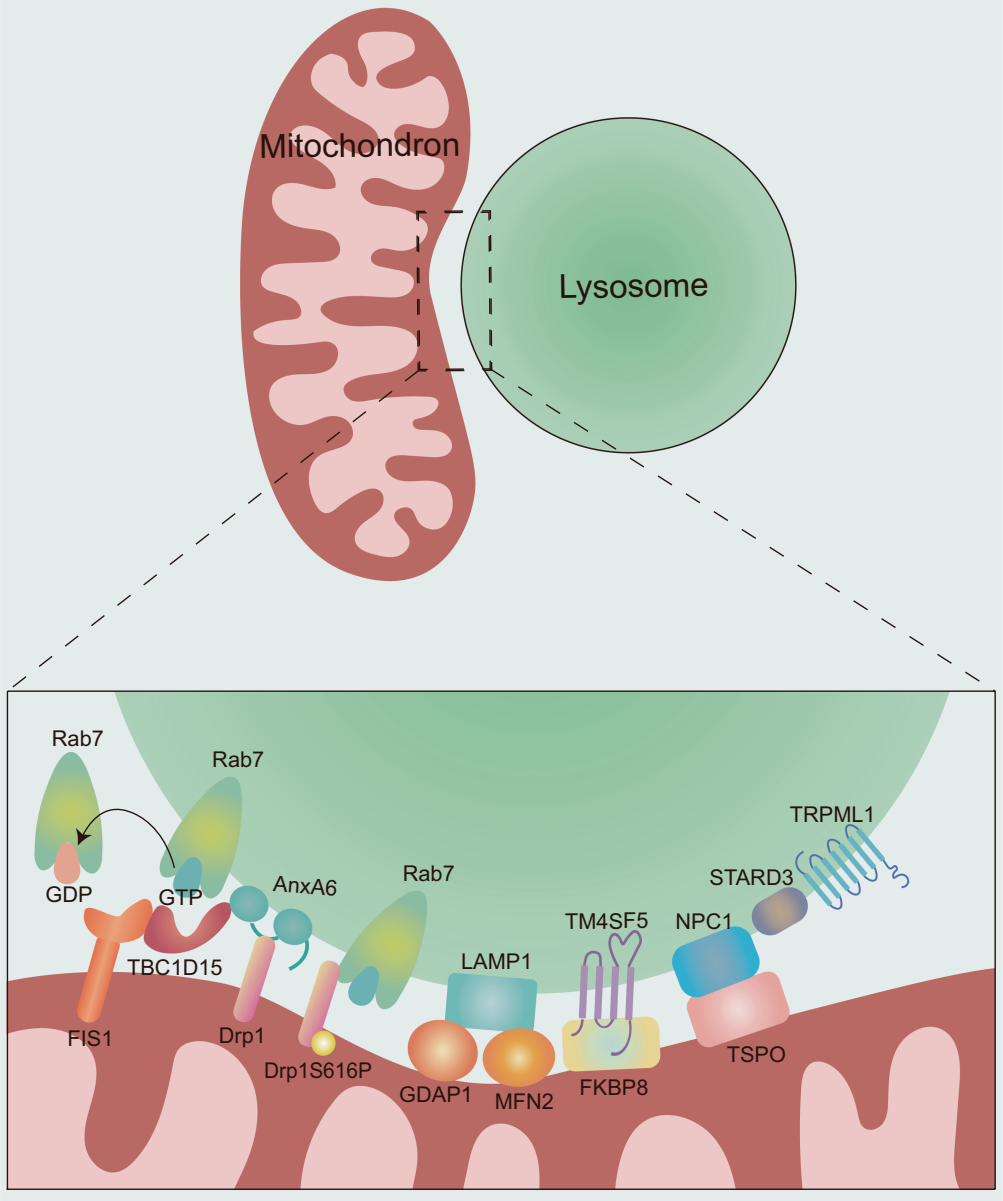
The molecular composition and regulation of MLCSs. The dynamics of MLCS are mechanically regulated by different tethering and untethering proteins. Rab7: the small GTPase Rab7 plays a critical role in the tethering and untethering of MLCSs. Active, GTP-bound Rab7 promotes MLCS formation, while the hydrolysis of GTP to GDP by TBC1D15 leads to untethering. TBC1D15: TBC1D15 is recruited to mitochondria via OMM protein FIS1, promoting MLCS untethering by hydrolyzing GTP-bound lysosomal Rab7 to GDP-bound state. AnxA6: AnxA6 plays a dual role in regulating MLCSs. First, it recruits TBC1D15 to lysosomes, facilitating Rab7 GTP hydrolysis and promoting MLCS untethering. Second, AnxA6 interacts with Drp1, whose phosphorylation (Drp1S616P) further promotes MLCS formation through its interaction with Rab7. GDAP1 and MFN2: the OMM proteins GDAP1 and MFN2 are involved in MLCS by interacting with LAMP1. TM4SF5: TM4SF5 translocates to the lysosomal membrane in response to extracellular glucose availability and contributes to MLCS formation through its interaction with FKBP8. NPC1 and TSPO: NPC1 and TSPO interact at MLCSs to support contact site formation. STARD3 and TRPML1: the late endosomal sterol-binding protein STARD3 and the lysosomal calcium efflux channel TRPML1 also play roles in MLCS regulation. However, the specific mechanisms by which these proteins contribute to MLCS dynamics remain unclear.

MLCS dynamics are regulated through different tethering and untethering protein machineries (Figure 2), which are crucial for the interaction between mitochondria and lysosomes, as well as our understanding of their potential influences on neurological diseases.

### 2.1 Rab7

Rab7, a small GTPase regulating lysosomal/late endosomal dynamics (Hutagalung and Novick, 2011; Markovinovic et al., 2022), modulates the dynamics of MLCS tethering and untethering (Wong et al., 2018). Active, GTP-bound lysosomal Rab7 induces MLCS formation. Hydrolysis of GTP-bound Rab7, which results in an inactive, GDP-bound state, is driven at contact sites by the GTPase-activating protein (GAP) TBC1 domain family member 15 (TBC1D15) (Peralta et al., 2010; Zhang et al., 2005), which is recruited to mitochondria via oligomers of the OMM protein FIS1 (Onoue et al., 2013), and GDP-bound Rab7 promote untethering dynamics (Wong et al., 2018). Indeed, the constitutively active Rab7 (Q67L) mutant which is unable to undergo Rab7 GTP hydrolysis leads to a higher percentage of lysosomes forming stable contacts and an extended duration of contact tetherings than wild-type Rab7 (Wong et al., 2018). Similarly, TBC1D15 GAP-domain mutants (D397A or R400K; Rab7 GTP hydrolysis inhibition) or FIS1 (LA) mutant (TBC1D15 mitochondrial recruitment disruption) resulted in increased contact durations (Wong et al., 2018). Furthermore, the small-molecule inhibitor ML-282, which competitively inhibits the Rab7 binding site at MLCSs, effectively reduced the number of these contacts (Wang K. et al., 2024). Recent findings highlight mitochondrial phosphorylated dynamic-related protein 1 (Drp1S616P) as a novel interorganellar tethering protein that localizes to the MLCS through interaction with Rab7, thereby enhancing crosstalk between these organelles (Che et al., 2022). Additionally, dephosphorylation of Drp1S616P mediated by the protein phosphatase 2A regulatory subunit B56γ inhibits the interaction between mitochondrial Drp1S616P and Rab7, thus suppressing mitochondria-lysosome crosstalk (Che et al., 2022). Understanding the intricate interplay between Rab7 and its binding partners may provide valuable insights into regulatory mechanisms and functional roles within the cell.

### 2.2 Transient receptor potential mucolipin 1

In addition to Rab7, the most studied regulatory factor, the endolysosomal, non-selective cation channel transient receptor potential mucolipin 1 (TRPML1), which modulates multiple biological processes, including endocytosis, exocytosis, lysosome reformation, lysosomal biogenesis, and autophagy (Qi et al., 2024), also regulates the dynamics of MLCSs (Peng et al., 2020). Cells expressing a dominant-negative TRPML1 mutant had a higher proportion of lysosomes in stable contact with mitochondria and a significantly longer minimum of contact duration (Peng et al., 2020). Future studies could further explore the specific regulatory modes of TRPML1 in MLCSs under different physiological and pathological conditions, as well as its interrelationships with other regulatory factors. This will facilitate a more comprehensive and in-depth understanding of the regulatory mechanism underlying the occurrence and function of MLCSs.

### 2.3 GDAP1 and MFN2

The OMM proteins ganglioside-induced differentiation-associated protein 1 (GDAP1) and mitofusin (MFN)2 which regulate mitochondrial division and fusion (Liesa et al., 2009; Wolf et al., 2022), respectively, are involved in MLCSs by interacting with LAMP1 (Cantarero et al., 2021; Pijuan et al., 2022). Pijuan et al. (2022) hypothesized that GDAP1 and MFN2 play coordinated roles in regulating MLCSs. Missense variants of GDAP1 and MFN2 do not decrease the expression of either protein but affect MLCSs (Pijuan et al., 2022). The discovery of GDAP1 and MFN2 as protein tethers between mitochondria and lysosomes highlights the relevance of mitochondrial dynamics for the MLCSs. Interestingly, missense mutations can occur within the catalytic domains of these two proteins, specifically in the GST-N domain of GDAP1 and the GTPase domain of MFN2 (Pijuan et al., 2022). Further investigation into how missense variants affect other domains of these proteins might provide valuable insights into the structure–function relationship of GDAP1 and MFN2 in the regulation of MLCSs.

### 2.4 Transmembrane 4 L six family member 5

Transmembrane 4 L six family member 5 (TM4SF5), a member of the transmembrane 4 L six family, can form numerous protein–protein complexes across various subcellular membranes with four transmembrane domains and two extracellular loops (Lee, 2015). TM4SF5 can translocate to the lysosomal membrane in response to extracellular glucose availability, facilitating MLCS formation through its interaction with mitochondrial FK506-binding protein 8 (FKBP8) (Kim et al., 2024). The formation of TM4SF5-mediated MLCSs was markedly reduced in hepatocytes expressing TM4SF5 mutants that were either palmitoylation-deficient (Pal^−^, C2/6/9/74/75/79/80/84/189A) or carried a mutation of the second transmembrane domain (C62A) (Kim et al., 2024). Rab7 inhibition reduced the levels of TM4SF5-enriched MLCS but did not completely abolish the formation of TM4SF5-dependent MLCSs following glucose treatment (Kim et al., 2024), suggesting that TM4SF5 may drive membrane interactions between mitochondria and lysosomes under conditions of extracellular glucose. Different proteins, including mitochondrial dynamics-related proteins, cholesterol transporters, and mitophagy receptors, may be localized in proximity to the TM4SF5-FKBP8 linkage site in TM4SF5-enriched MLCSs (Kim et al., 2024). The formation and aggregation of these protein complexes mediated by lysosomal TM4SF5 highlight the biological importance of TM4SF5-enriched MLCS, which appear to play a crucial role in the mitochondrial dynamics, transfer of cholesterol from lysosomes to mitochondria and mitophagy in the context of extracellular glucose.

### 2.5 Others

AnxA6, the largest member of the annexin family, is involved in regulating various cellular processes, including endocytic and exocytic pathways, cholesterol homeostasis, and the formation of multifactorial signaling complexes (Garcia-Melero et al., 2016). In addition to these roles, AnxA6 is associated with membrane contact site (MCS) formation (Garcia-Melero et al., 2016). It has been shown to reduce GTP-bound Rab7 levels by recruiting TBC1D15 to lysosomes, also interacts with Drp1 and may play a scaffolding role in Rab7/TBC1D15- and Drp1-dependent MLCS dynamics (Chlystun et al., 2013; Meneses-Salas et al., 2020). Electron microscopy results indicated that the late endosomal sterol-binding protein STARD3 (also called MLN64) can be located at the mitochondria-lysosome interface, suggesting that STARD3 may play a key role in mediating or stabilizing the interaction between these two organelles. Specifically, the study by Höglinger et al. demonstrated that STARD3 depletion substantially reduces the proportion of lysosomes that form contact sites with mitochondria (Höglinger et al., 2019). Moreover, the interaction between the lysosomal Niemann-Pick type C (NPC)1 protein and the mitochondrial translocator protein (TSPO) promotes the formation of MLCSs (Lin et al., 2023). Furthermore, the mammalian target of rapamycin complex 1 (mTORC1), a vital cellular nutrient and stress receptor, plays a crucial role in maintaining cellular homeostasis (Su and Zheng, 2021). It has been shown that mTORC1 activation inhibits the interaction between lysosomal NPC1 and mitochondrial TSPO, thereby modulating MLCS formation (Lin et al., 2023). This highlights that mTORC1 could regulate the balance between cellular signaling and organelle dynamics, influencing contact site formation under different metabolic or stress conditions. Interestingly, recent finding indicates that the absence of NPC1 leads to an increase in STARD3-dependent MLCSs, suggesting that the loss of NPC1 may alter STARD3-dependent mechanisms at MLCSs (Höglinger et al., 2019).

Collectively, these findings reveal a complex network of proteins that regulate MLCS dynamics, each contributing in distinct ways to tethering or untethering processes. While the intricate interplay of some proteins highlights the complexity of mitochondria-lysosome interactions, the specific regulatory mechanisms of others remain poorly understood, emphasizing the need for further investigation. Concurrently, future studies should focus on elucidating the biological implications of distinct MLCS subtypes formed through various tethering mechanisms.

## 3 Functions of mitochondria-lysosome contact sites

Membrane domains formed at MLCSs serve as critical hubs for coordinating communication between mitochondria and lysosomes, influencing cellular metabolic processes, and maintaining overall cellular homeostasis (Lackner, 2019).

### 3.1 Mitochondrial dynamics and network

Mitochondria are highly dynamic organelles that undergo continuous fission and fusion to maintain functional networks and mitochondrial homeostasis (Chan, 2006; Kyriakoudi et al., 2021). In mammals, mitochondrial fission is regulated by Drp1 and other members of the dynamin family of large GTPases (Otera et al., 2010), whereas fusion is controlled by the OMM large GTPases MFN1 and MFN2, along with optic atrophy 1 in the inner mitochondrial membrane (Cipolat et al., 2004). MLCSs regulate the mitochondrial network by marking mitochondrial fission sites and controlling the rate of fission events (Kleele et al., 2021; Wong et al., 2018). There are two distinct types of mitochondrial fission: midzone division (within the central 50%) and peripheral division (less than 25% from the tip). Both types of fission are mediated by Drp1, but the mechanisms that regulate them differ. Pre-constriction, which involves the endoplasmic reticulum (ER) and actin, along with the mitochondrial fission factor (MFF), specifically governs midzone fission. In contrast, peripheral fission is regulated by the OMM protein FIS1. Midzone division facilitates mitochondrial proliferation, whereas peripheral division promotes the removal of damaged mitochondria by fission into smaller units for mitophagy (Kleele et al., 2021). These distinct molecular mechanisms explain how cells can independently regulate fission events, leading to different mitochondrial fates. A decrease in extracellular matrix stiffness can promote excessive mitochondria-lysosome contacts, which in turn exacerbate mitochondrial peripheral division. This leads to the accumulation of ROS within the mitochondria, causing mitochondrial oxidative stress and ultimately disrupting cellular homeostasis (Wang K. et al., 2024). Rab7 (Q67L) mutant and TBC1D15 (D397A, R400K) mutants resulted in reduced mitochondrial fission rates and an increase in overly elongated mitochondrial networks (Wong et al., 2018). Moreover, TBC1D15-mediated MLCS untethering plays a regulatory role in asymmetric mitochondrial fission (Sun et al., 2022) because TBC1D15 interacts with Drp1 through its C-terminal domain (574–624), recruiting Drp1 to MLCSs and facilitating asymmetric fission (Sun et al., 2022). Qiu et al. developed an optogenetic system for controlling mitochondrial fission through a light-induced MLCS. This system allows spatiotemporal control and reversibility of MLCS formation (Qiu et al., 2022), offering the potential for the investigation of mitochondrial fission and the treatment of mitochondrial diseases. Moreover, MLCSs mark and regulate inter-mitochondrial contact untethering events, with the formation and untethering of these contacts regulated by MFN1/2 and Drp1 GTP hydrolysis, respectively (Wong et al., 2019b). These insights underscore the pivotal role of MLCSs in orchestrating mitochondrial network dynamics and highlight its potential as a therapeutic target for correcting mitochondrial dysfunction in disease.

### 3.2 Lysosomal dynamics

Beyond the canonical role of lysosomes in cellular waste management, lysosomes function as signaling hubs involved in nutrient sensing, immune cell signaling, metabolism, and membrane repair, allowing them to detect, adapt, and react to fluctuations in substrate metabolism to preserve cellular function (Perera and Zoncu, 2016; Trivedi et al., 2020). Lysosomal localization is a dynamically regulated process that is a critical determinant of lysosomal function (Ballabio and Bonifacino, 2020). Thus, elucidating the regulation of lysosomal networks is vital for investigating cellular homeostasis and disease mechanisms. In addition to GTP-bound, active Rab7, which regulates lysosomal transport, fusion, and maturation (Hutagalung and Novick, 2011; Wong et al., 2018), lysosomal dynamics are also strongly influenced by Rab7 effector proteins that preferentially interact with GTP-bound Rab7 on lysosomal membranes. Notable among these are Rab7-interacting lysosomal protein and the protein FYVE and coiled-coil domain containing 1 (Jordens et al., 2001; Pankiv et al., 2010), which facilitate retrograde and anterograde microtubule transport, respectively, as well as the homotypic fusion and protein sorting complex, which facilitates lysosomal tethering and fusion (Balderhaar and Ungermann, 2013). MLCSs serve as a platform for mitochondria-localized proteins to regulate lysosomal dynamics by modulating Rab7 GTP hydrolysis. Specifically, mitochondrial TBC1D15 promotes the hydrolysis of Rab7 GTP at the MLCS, leading to untethering of these contacts (Wong et al., 2018) and subsequent release of Rab7 effector proteins from GTP-bound Rab7 and lysosomal membranes, thereby influencing lysosomal dynamics. Intriguingly, the mitochondrial oligomeric Mid51/Fis1 complex links during this process Drp1 and the Rab7 GTP hydrolysis machinery to regulate inter-lysosomal untethering and network dynamics (Wong et al., 2022). Dysregulation of this pathway may impair lysosomal network dynamics, including interruption of lysosomal movement, distribution, and cargo transport (Wong et al., 2022). Furthermore, reduced TBC1D15 expression can result in the accumulation of abnormal MLCSs, leading to acidification defects and lysosomal enlargement (Yu et al., 2020). A possible reason for these alterations is the disruption of metabolic exchange processes by abnormal MLCSs, leading to excessive accumulation of substances and subsequent lysosomal dysfunction. Recent study has shown that soft substrates microenvironment can stimulate an increase in lysosomal activity, resulting in enhanced lysosomal motility (Wang K. et al., 2024). This, in turn, increases the frequency of MLCSs, which can subsequently impact lysosomal dynamics (Wang K. et al., 2024). The finding highlights the reciprocal relationship between lysosomal dynamics and MLCSs, which act together to maintain cellular homeostasis.

### 3.3 Substance exchanges

Mitochondria and lysosomes are crucial for the distribution of various ions and metabolites, including calcium, cholesterol, and iron (Cisneros et al., 2022; Han et al., 2017). MLCS provide a platform for substance exchange between two organelles, which plays an important role in maintaining ions and metabolic homeostasis.

#### 3.3.1 Calcium

Both mitochondria and lysosomes play major roles in cellular calcium homeostasis. Calcium is transported into mitochondria to facilitate intracytoplasmic calcium removal and to stimulate metabolic processes, including ATP synthesis, ROS production, and apoptosis (Rosencrans et al., 2021; Todkar et al., 2017). While the regulatory role of mitochondria in calcium homeostasis is well established, the recognition of lysosomes as calcium stores has only recently emerged. Lysosomal calcium signaling regulates various cellular processes, including autophagy, membrane fusion, endocytic membrane trafficking, and cell death (Ballabio and Bonifacino, 2020; Todkar et al., 2017). A recent study by Peng et al. utilized high spatial and temporal resolution live-cell microscopy to show that the lysosomal calcium efflux channel TRPML1 preferentially meditates lysosomal calcium transfer to mitochondria at the MLCSs, modulated by the voltage-dependent anion channel 1 (VDAC1) and mitochondrial calcium uniporter (MCU) (Peng et al., 2020). MLCSs may serve as a platform for contact-dependent calcium transfer from lysosomes to mitochondria, promoting downstream calcium-dependent mitochondrial functions such as oxidative phosphorylation, mitochondrial fission, trafficking, and ROS signaling. This prevents the activation of the mitochondrial permeability transition pore due to excess or sustained increases in mitochondrial calcium concentrations, which can trigger mitochondrial swelling, ∆ψm collapse, bioenergetic failure, and ultimately necrotic cell death (Pedersen et al., 2021). Notably, TRPML1 is activated by an increase in ROS levels, resulting in calcium efflux from lysosomes and nuclear translocation of transcription factor EB (TFEB). If this process fails, ROS accumulation leads to a loss of mitochondrial membrane potential and fragmentation, emphasizing the bidirectional regulation of mitochondrial function by TRPML1 (Todkar et al., 2017). A broader understanding of calcium regulation mechanisms in the crosstalk between mitochondria and lysosomes will enhance our understanding of their role in cellular homeostasis and disease. Further research is required to elucidate the mechanism of calcium-dependent translocation of MLCSs.

#### 3.3.2 Cholesterol

Cholesterol is essential for maintaining the structural integrity of cell membranes and for supporting cellular functions (Chen et al., 2023). Mitochondria acquire cholesterol from distinct subcellular pools via various pathways and convert it into sterol metabolites, which are crucial for physiological processes (Juhl et al., 2021; Kennedy et al., 2012). Emerging research has highlighted MLCSs as a vital site for cholesterol transfer between lysosomes and mitochondria, which is mediated by specific protein interactions. NPC1 is a large transmembrane protein located in the limiting membrane of late endocytic organelles, while NPC2 is a small soluble luminal protein that transfers cholesterol from intra-lysosomal vesicle membranes to the N-terminal domain of NPC1 (Infante et al., 2008). Together, they play a pivotal role in the cholesterol regulation. One study highlighted that the lysosomal protein NPC2 is involved in transferring cholesterol from lysosomes to mitochondria in fibroblasts *in vitro*, underscoring the significance of MLCSs in cellular cholesterol dynamics (Juhl et al., 2021). Additionally, mTORC1, whose activity is regulated by Rab7 (Li et al., 2010), located on lysosomes senses cholesterol signals through intercellular contacts, thereby regulating the interactions of cholesterol transport proteins (Zhu and Wang, 2020). Subsequent study has revealed that mTORC1 mediates the transport of cholesterol from lysosomes to mitochondria by regulating the interaction between NPC1 and TSPO, which triggers lysosomal membrane permeabilization and mitochondria-dependent necroptosis-mediated hepatotoxicity through the accumulation of inter-organelle free cholesterol (Lin et al., 2023). Moreover, TM4SF5 translocates to lysosomes upon extracellular glucose availability, where it interacts with NPC1 and free cholesterol to further drive cholesterol transport to mitochondria (Kim et al., 2024). As an emerging research focus, cholesterol transport via MLCSs offers new insights into cellular homeostasis. Unpacking the molecular mechanisms underlying this transfer will enhance our understanding of cholesterol-related cell signaling and interorganelle communication, providing potential therapeutic targets.

#### 3.3.3 Iron

Mitochondria serve as a major reservoir of cellular iron, containing 20–50% of the total iron within cells (Ward and Cloonan, 2019). Iron uptake by mitochondria is critical for essential processes, such as heme synthesis and iron–sulfur cluster biogenesis, which support key cellular functions, including the electron transport chain, metabolic conversions, and protein synthesis (Giacomello et al., 2020; Pedersen et al., 2021). Concurrently, iron is present at micromolar concentrations within the lysosomal lumen and is released from lysosomes into the cytoplasm via the TRPML1 (Trivedi et al., 2020). Owing to these functions, iron levels are tightly regulated. Recent studies have shown that MLCSs facilitate the transfer of iron from lysosomes to mitochondria, potentially via TRPML1 (Khalil et al., 2017; Li et al., 2024). These interactions further underscore the vital role of MLCSs in coordinating cellular iron distribution and metabolic balance.

#### 3.3.4 Amino acids

In addition to calcium, cholesterol, and iron, MLCSs also play a critical role in maintaining cellular amino acid homeostasis. Recent research suggests that abnormal MLCSs can disrupt the amino acid balance, marked by amino acid accumulation in lysosomes and amino acid deficiency in mitochondria (Peng et al., 2023). This imbalance underscores the functional importance of MLCSs in coordinating organelle-specific amino acid profiles to sustain cellular functions. Restoring stable MLCSs partially recovered amino acid levels within the entire cell, as well as in mitochondria and lysosomes (Peng et al., 2023), suggesting a regulatory role for MLCSs in amino acid distribution.

Overall, continued research on MLCSs and substance exchanges is likely to provide new insights into the fundamental biology of cellular metabolism.

### 3.4 Mitophagy

Although MLCS represents a novel mechanism of organelle crosstalk that is independent of mitophagy, studies have indicated that these contacts are linked to mitophagy (Juhl et al., 2021; Kim et al., 2024; Wong et al., 2019a). Proximity labeling revealed the molecular clustering of Drp1S616P and specific mitophagy receptors (BCL2L13, PHB2, AMBRA1, OPA1, and VDAC1) in TM4SF5-enriched MLCSs, promoting mitochondrial fission and autophagy (Kim et al., 2024). Indeed, the formation of TM4SF5-enriched MLCSs upon glucose repletion reduced mitochondrial length. Notably, mitochondrial Drp1S616P interacts with Rab7 at the MLCS to mediate PINK1-Parkin-dependent mitophagy in hepatocellular carcinoma cells (Che et al., 2022). Furthermore, the calcium regulation by MLCSs can activate the TFEB pathway (TFEB nuclear translocation through calcineurin- and Rag GTPase-dependent pathways) and downstream protective cellular responses, including autophagy (Giamogante et al., 2024). Specifically, MLCSs mark mitochondrial peripheral fission sites, prompting damaged material to segregate into smaller mitochondria for mitophagy (Kleele et al., 2021). Peripheral fission is associated with oxidative stress, cellular damage, and high energy demands. Interestingly, mitochondrial dysfunction—manifested by elevated mitochondrial calcium levels, increased ROS, reduced membrane potential, and lower pH—along with the recruitment of autophagic machinery, such as the accumulation of Parkin on mitochondria, precedes peripheral fission (Kleele et al., 2021). These features link peripheral fission to mitophagy, PINK1-mediated mitochondrial turnover, and Parkin-regulated autophagy, while also providing localization cues for fission events at the periphery. Although research on MLCSs and mitophagy has provided initial insights, future studies should explore how proteins and signaling molecules regulating MLCSs affect the recognition of damaged mitochondria during the early stages of mitophagy.

### 3.5 RNA translation

Recent studies have highlighted the emerging role of MLCSs in regulating localized RNA translation (Cioni et al., 2019; Fenton et al., 2024). A key finding revealed that Rab7a-positive late endosomes, which carry RNA, form contacts with mitochondria in axons, acting as translation platforms (Cioni et al., 2019). This translation at MLCSs is crucial for maintaining axonal function and mitochondrial integrity, as it enables the synthesis of nascent proteins required for axonal mitochondrial function. In addition to facilitating the trafficking of mRNAs, MLCSs provide a spatially regulated environment for the translation machinery. The localized translation at MLCSs ensures that mitochondrial proteins are synthesized in close proximity to mitochondria, promoting efficient protein import into mitochondria. Another study showed that late endosomes and lysosomes in neuronal axons and dendrites localize the key translational regulator fragile X messenger ribonucleoprotein particles, which are enriched in ribosome structures, to the mitochondrial midzone in a Rab7-dependent manner (Fenton et al., 2024), serving as a platform for the translation of MFF to promote mitochondrial fission at the mitochondrial midzone.

In summary, MLCSs fulfill diverse functions, are indispensable for various aspects of cellular biology, playing critical roles in maintaining cellular homeostasis, and support physiological processes. Continued research in this field is essential to fully understand the potential of MLCSs and their significance in biological systems and disease mechanisms.

## 4 Mitochondria-lysosome contact sites and neurological disorders

Various neurological diseases are associated with mitochondrial and lysosomal dysfunction (Ballabio and Bonifacino, 2020; Bonam et al., 2019), indicating that abnormal interactions between these two organelles may lead to neuronal damage and, ultimately, neurological diseases. After the dynamic formation of these contacts in human neuronal soma, axons, and dendrites has first been described, many studies have investigated the relationship between MLCS impairments and various neurological diseases (Table 1; Kim et al., 2021).

**Table 1:** Genes and proteins involved in MLCS-related neurological disorders.

Disease		Key genes or proteins	Effect on MLCSs	Potential pathophysiological implication	References
PD	–	GBA1	Prolonged MLCS duration, disruption of mitochondrial distribution and function	GBA1 mutation causes defective TBC1D15 regulation, impairing mitochondrial function and dynamics, contributing to GBA1-associated PD pathogenesis	Kim et al. (2021)
	–	α-synuclein (A30P and A53T)	Disruption of MLCS tethering, elevated local cytosolic calcium levels, activation of the TFEB pathway	Disruption of MLCS tethering and subsequent elevated local cytosolic calcium levels lead to calcineurin activation, TFEB dephosphorylation, and cytoprotective responses, which counteract neurodegenerative processes at an early presumably protective stage	Giamogante et al. (2024)
	–	Mid51 (R169W)	Inhibition of inter-lysosomal untethering events mediated by MLCSs, dysregulation of lysosomal network dynamics	Aberrant lysosomal network dynamics play a role in neurodegeneration	Wong et al. (2022)
	–	Parkin	reduced number of MLCSs, accumulation of amino acids in lysosomes with a corresponding deficiency in mitochondria	Parkin mutations destabilize Rab7, affecting MLCS formation and leading to disrupted amino acid metabolism	Peng et al. (2023)
CMT disease	CMT2A	Mfn2 (T105M)	Impaired MLCS-regulated inter-mitochondrial untethering, decreased mitochondrial network motility	Mutations impair MLCS regulation, causing mitochondrial network defects that contribute to disease progression	Wong et al. (2023) and Wong et al. (2019a)
	CMT2C	TRPV4 (R269H)			
	CMT2B	Rab7 (V162M)			
		Rab7 (K157N, L129F, V162M, and N161T)	Disruption of MLCS-regulated mRNA translation essential for mitochondrial and axonal integrity	Impairment of mitochondrial function and axonal integrity	Cioni et al. (2019)
	–	GDAP1 (deficiency)	Reduced cellular glutathione levels, lysosomal membrane defects, defective MLCSs	GDAP1 deficiency or mutations affect redox homeostasis, disrupt MLCSs, and impact lysosomal and mitochondrial function	Cantarero et al. (2021)
	–	GDAP1 (p.Thr157Pro)	Increased number of MLCSs, a hyperfissioned mitochondrial network		Cantarero et al. (2023)
	–	GDAP1 (p.Arg161His)	Decreased number of MLCSs, elongated mitochondria		Cantarero et al. (2023)
LSD	MLIV	TRPML1	Changes in MLCSs and dysfunction of contact-dependent mitochondrial calcium uptake	Loss of TRPML1 function impairs MLCSs and mitochondrial calcium regulation, promoting disease onset	Peng et al. (2020)
	NPC disease	NPC1	Impaired transport of cholesterol mediated by the ER-endocytic organelle MCS, increased number of MLCSs in response to lysosomal cholesterol accumulation	MLCSs may play a compensatory role by supporting mitochondrial cholesterol accumulation, affecting mitochondrial function	Höglinger et al. (2019)
Epilepsy	–	TBC1D15	Abnormalities in MLCS, excessive calcium influx from lysosomes into mitochondria, mitochondrial dysfunction	Overexpression of TBC1D15 protects against neuronal injury by modulating MLCSs and reducing mitochondrial dysfunction in epilepsy	Xie et al. (2024)

### 4.1 Parkinson’s disease

PD is a multisystem neurologic disorder characterized by Lewy bodies, progressive loss of dopaminergic neurons in the substantia nigra pars compacta, and a unique motor phenotype (parkinsonism) (Ye et al., 2023). Mitochondrial and lysosomal dysfunctions are genetically and functionally associated with PD (Burbulla et al., 2017; Nguyen et al., 2019). Kim et al. investigated the direct homeostatic relationship between these two organelles and found that neurons with *GBA1* (the gene encoding the lysosomal enzyme *β*-glucocerebrosidase, which catalyzes the hydrolysis of glucosylceramide into glucose and ceramide) mutations found in patients with PD exhibited prolonged MLCS duration and consequent disruption of mitochondrial distribution and function (oxidative phosphorylation and ATP levels) due to defective modulation of the untethering protein TBC1D15 (Kim et al., 2021). Thus, GBA1 can be considered an upstream regulator of mitochondrial function and dynamics in midbrain dopaminergic neurons. Importantly, mitochondrial dysfunction in these cells can be partially rescued by expressing TBC1D15, highlighting the potential role of MLCSs in the pathogenesis of GBA1-associated PD (Kim et al., 2021). A recent study has shown that the overexpression of wild-type or PD-associated A30P and A53T *α*-synuclein mutants significantly disrupts MLCS tethering (Giamogante et al., 2024). This disruption impedes mitochondrial calcium uptake after lysosomal calcium release, leading to localized increases in cytosolic calcium levels (Giamogante et al., 2024). Consequently, this may trigger calcineurin activation and TFEB dephosphorylation, which in turn regulate the TFEB pathway and downstream cytoprotective responses, such as autophagy and lysosome biogenesis, to counteract neurodegenerative processes at early presumably protective response. However, at a later stage, the decreased expression of TFEB in the nuclear fraction (sequestered into the cytoplasm by α-Syn) resulted in the impairment of autophagy. Furthermore, the oligomerization domain mutant Mid51 (R169W) has recently been identified as a potential candidate genetic variant for PD as it can inhibit inter-lysosomal untethering events mediated by MLCSs, resulting in the dysregulation of lysosomal network dynamics (Wong et al., 2022). Moreover, in dopaminergic neurons derived from induced pluripotent stem cells of patients with Parkin-associated PD, Parkin mutations destabilize active Rab7, leading to reduced numbers of MLCSs (Peng et al., 2023). Subcellular metabolomics analysis revealed an accumulation of amino acids in lysosomes and a corresponding amino acid deficiency in mitochondria (Peng et al., 2023), reflecting disrupted amino acid homeostasis. Interestingly, TBC1D15 knockdown partially restored MLCSs and improved cellular and subcellular amino acid profiles (Peng et al., 2023). These findings suggest that targeting the regulatory mechanisms of MLCSs may offer new therapeutic opportunities in PD, particularly for addressing amino acid imbalances in Parkin-associated PD. Overall, these findings highlight the potential of MLCSs as a therapeutic target for broader intervention strategies in PD.

### 4.2 Charcot–Marie–Tooth disease

CMT disease is a genetically heterogenous group of primary peripheral neuropathies characterized by progressive distal muscle atrophy and weakness, sensory loss, and limb deformities (Volodarsky et al., 2021). Mutations in CMT-related genes lead to either demyelination or axonal degeneration (Petkovic et al., 2021), and numerous genetic variants have been identified as the underlying causes of different CMT subtypes (Young et al., 2008). Understanding the distinct pathogenic mechanisms associated with each CMT subtype is essential for identifying new molecular targets and developing novel therapeutic strategies. Various mutations linked to CMT2 cause defects in mitochondrial dynamics, underscoring the importance of MLCSs in mitochondrial network regulation and disease progression. For instance, mutations such as MFN2 (T105M) in CMT2A (Züchner et al., 2004), Rab7 (V162M) in CMT2B (Houlden et al., 2004), and TRPV4 (R269H) in CMT2C (Auer-Grumbach et al., 2010) impair MLCS-regulated inter-mitochondrial untethering, leading to decreased mitochondrial network motility (Wong et al., 2023; Wong et al., 2019b). Recent study has shown that Rab7a mutations (K157N, L129F, V162M, and N161T), which are associated with CMT2B, disrupt the translation of mRNAs critical for mitochondrial and axonal integrity, leading to a significant reduction in axonal protein synthesis, impairing mitochondrial function, and compromising axonal vitality (Cioni et al., 2019). Mutations in *GDAP1*, a gene encoding the enzyme glutathione S-transferase (Cantarero et al., 2021), are associated with various CMT phenotypes, including autosomal dominant axonal CMT (CMT2K), autosomal recessive axonal CMT (AR-CMT2K), and autosomal recessive demyelinating CMT (CMT4A) (Gonzalez-Sanchez et al., 2019). GDAP1 deficiency reduces cellular glutathione levels, leads to lysosomal membrane defects, and results in defective MLCSs (Cantarero et al., 2021). Glutathione-reduced ethyl ester supplementation can effectively rescue lysosomal and mitochondrial network defects resulting from abnormal MLCSs (Cantarero et al., 2021), suggesting that GDAP1 may act as a tether, regulating or sensing the redox state of the MLCSs. However, the exact role of MLCSs in the redox regulation in CMT disease remains to be elucidated. Recent studies have revealed the phenotypic heterogeneity of GDAP1 variants based on their effects on MLCSs. Variants in the *α*-loop interaction domain exhibit distinct functional consequences; the dominant variant p.Thr157Pro increases the number of MLCSs, causing mitochondrial hyperfission, whereas the recessive variant p.Arg161His decreases the number of MLCSs, resulting in elongated mitochondria (Cantarero et al., 2023). Additionally, cases of co-inheritance of pathogenic variants in *GDAP1* and *MFN2*—two genes with overlapping functions in MLCS regulation—suggest an additive effect on axonal CMT phenotypes (Anghelescu et al., 2017; Kostera-Pruszczyk et al., 2014), further supporting the collaborative role of GDAP1 and MFN2 in MLCS regulation.

### 4.3 Lysosomal storage diseases

LSD, a group of hereditary disorders leading to lysosomal substrate accumulation and lysosomal dysfunction, are characterized by progressive multisystemic phenotype that includes neurodegeneration (Frosch and Prinz, 2024). A recent study found dynamic changes in MLCSs and contact-dependent mitochondrial calcium uptake dysfunction in fibroblasts of patients with Mucolipidosis type IV, an autosomal recessive LSD caused by loss-of-function mutations in TRPML1 (Peng et al., 2020). Thus, in Mucolipidosis type IV, the loss of TRPML1 function may promote disease onset through impairment of the aforementioned pathways, further confirming the convergence of mitochondrial and lysosomal dysfunctions in this disease. NPC disease is an autosomal recessive neurodegenerative LSD caused by mutations in NPC1 or NPC2 and is characterized by excessive accumulation of cholesterol in lysosomes (Colaco et al., 2020; Martello et al., 2020; Zhang et al., 2002). NPC1 interacts with the ER-localized protein Gramd1b, which connects the ER-endolysosomal MCS to regulate cholesterol release (Höglinger et al., 2019). In NPC1-deficient cells, cholesterol transport mediated by the ER-endocytic organelle MCS is impaired, leading to increased numbers of MLCSs dependent on STARD3 in response to lysosomal cholesterol accumulation (Höglinger et al., 2019). This indicates that MLCSs may play a compensatory role, providing a possible mechanism for mitochondrial cholesterol accumulation and dysfunction observed in NPC. Future studies are needed to elucidate whether changes in organelle contacts in NPC disease affect previously observed mitochondrial phenotypes (e.g., ATP production and mitochondrial networks) (Wos et al., 2016).

### 4.4 Epilepsy

Epilepsy is a complex neurological disorder characterized by a persistent tendency for spontaneous seizures (Wang H. et al., 2024). Recent studies have elucidated the crucial role of mitochondrial dysfunction in the pathophysiology of epilepsy, emphasizing that mitochondrial calcium overload is a significant contributor to neuronal damage (Xie et al., 2024; Xu et al., 2024). Our recent research has demonstrated that seizures can lead to abnormalities in MLCS, resulting in excessive calcium influx from lysosomes into mitochondria, subsequently causing mitochondrial calcium overload, which then results in mitochondrial dysfunction characterized by decreased mitochondrial membrane potential and increased ROS levels (Xie et al., 2024). Overexpression of TBC1D15 effectively regulates MLCSs and alleviates mitochondrial calcium overload and associated dysfunction (Xie et al., 2024). This protective mechanism shields neurons from injury, highlighting the potential of MLCSs as a therapeutic target for treating neuronal damage in epilepsy.

## 5 Conclusion and perspective

The study of organelle interactions has long been a fundamental aspect of cell biology, particularly concerning interactions within the endosomal system, which have been known for decades. The recently discovered contacts between mitochondria and lysosomes represent an exciting new frontier in this field. While much of the earlier research has utilized yeast as a model system (Elbaz-Alon et al., 2014), current investigations have increasingly focused on mammalian cells and tissues (Cisneros et al., 2022; Pijuan et al., 2022). In mammalian cells, the contact sites between mitochondria and lysosomes are regulated by various tethering/untethering proteins, which in turn modulate mitochondrial and lysosomal dynamics, as well as substance transfer, underscoring the complex crosstalk between these two organelles.

Neurons are particularly sensitive to disruptions in mitochondrial and lysosomal functions, suggesting that MLCSs may serve as a critical hub for neuronal homeostasis (Cantarero et al., 2021; Haidar and Timmerman, 2017). The exploration of these contact sites might help elucidate the simultaneous dysfunction of these organelles observed in various neurological diseases, including PD, CMT, and LSD.

The initial mechanisms underlying lysosomal and mitochondrial dysfunctions in neurological diseases remain debatable. Future research utilizing diverse techniques and methodologies is necessary to elucidate the molecular mechanisms and regulatory pathways involved in MLCS function in different cell types. Understanding how these mechanisms adapt to varying metabolic and signaling states within cells will enhance our understanding of the pathophysiological events underlying neurological diseases, particularly in identifying new biomarkers and potential pharmacological targets.

## References

[ref1] Anghelescu C. Francou B. Cardas R. Guiochon-Mantel A. Aubourg P. Servais L.. (2017). Targeted exomes reveal simultaneous and mutations in a severe Charcot-Marie-Tooth disease type 2 phenotype. Eur. J. Neurol. 24, e15–e16. doi: 10.1111/ene.13250, PMID: 28211244

[ref2] Auer-Grumbach M. Olschewski A. Papic L. Kremer H. McEntagart M. E. Uhrig S.. (2010). Alterations in the ankyrin domain of TRPV4 cause congenital distal SMA, scapuloperoneal SMA and HMSN2C. Nat. Genet. 42, 160–164. doi: 10.1038/ng.508, PMID: 20037588 PMC3272392

[ref3] Balderhaar H. J. K. Ungermann C. (2013). CORVET and HOPS tethering complexes - coordinators of endosome and lysosome fusion. J. Cell Sci. 126, 1307–1316. doi: 10.1242/jcs.107805, PMID: 23645161

[ref4] Ballabio A. Bonifacino J. S. (2020). Lysosomes as dynamic regulators of cell and organismal homeostasis. Nat. Rev. Mol. Cell. Biol. 21, 101–118. doi: 10.1038/s41580-019-0185-4, PMID: 31768005

[ref5] Bonam S. R. Wang F. J. Muller S. (2019). Lysosomes as a therapeutic target. Nat. Rev. Drug Discov. 18, 923–948. doi: 10.1038/s41573-019-0036-1, PMID: 31477883 PMC7097195

[ref6] Burbulla L. F. Song P. P. Mazzulli J. R. Zampese E. Wong Y. C. Jeon S.. (2017). Dopamine oxidation mediates mitochondrial and lysosomal dysfunction in Parkinson's disease. Science 357, 1255–1261. doi: 10.1126/science.aam9080, PMID: 28882997 PMC6021018

[ref7] Cantarero L. Garcia-Vargas G. Hoenicka J. Palau F. (2023). Differential effects of Mendelian GDAP1 clinical variants on mitochondria-lysosome membrane contacts sites. Biol. Open 12:bio059707. doi: 10.1242/bio.059707, PMID: 36912213 PMC10110396

[ref8] Cantarero L. Juárez-Escoto E. Civera-Tregón A. Rodríguez-Sanz M. Roldán M. Benítez R.. (2021). Mitochondria-lysosome membrane contacts are defective in GDAP1-related Charcot-Marie-Tooth disease. Hum. Mol. Genet. 29, 3589–3605. doi: 10.1093/hmg/ddaa243, PMID: 33372681 PMC7823109

[ref9] Chan D. C. (2006). Mitochondria: dynamic organelles in disease, aging, and development. Cell 125, 1241–1252. doi: 10.1016/j.cell.2006.06.010, PMID: 16814712

[ref10] Che L. Wu J. S. Xu C. Y. Cai Y. X. Lin J. X. Du Z. B.. (2022). Protein phosphatase 2A-B56gamma-Drp1-Rab7 signaling axis regulates mitochondria-lysosome crosstalk to sensitize the anti-cancer therapy of hepatocellular carcinoma. Biochem. Pharmacol. 202:115132. doi: 10.1016/j.bcp.2022.115132, PMID: 35697120

[ref11] Chen Q. X. Jin C. Z. Shao X. T. Guan R. L. Tian Z. Q. Wang C. R.. (2018). Super-resolution tracking of mitochondrial dynamics with an iridium(III) Luminophore. Small 14:e1802166. doi: 10.1002/smll.201802166, PMID: 30350549

[ref12] Chen W. J. Xu J. Z. Wu Y. D. Liang B. Yan M. Z. Sun C. D.. (2023). The potential role and mechanism of circRNA/miRNA axis in cholesterol synthesis. Int. J. Biol. Sci. 19, 2879–2896. doi: 10.7150/ijbs.84994, PMID: 37324939 PMC10266072

[ref13] Chlystun M. Campanella M. Law A. L. Duchen M. R. Fatimathas L. Levine T. P.. (2013). Regulation of mitochondrial morphogenesis by annexin A6. PLoS One 8:e53774. doi: 10.1371/journal.pone.0053774, PMID: 23341998 PMC3544845

[ref14] Cioni J. M. Lin J. Q. Holtermann A. V. Koppers M. Jakobs M. A. H. Azizi A.. (2019). Late endosomes act as mRNA translation platforms and sustain mitochondria in axons. Cell 176:e15, 56–72.e15. doi: 10.1016/j.cell.2018.11.030, PMID: 30612743 PMC6333918

[ref15] Cipolat S. de Brito O. M. Dal Zilio B. Scorrano L. (2004). OPA1 requires mitofusin 1 to promote mitochondrial fusion. Proc. Natl. Acad. Sci. U. S. A. 101, 15927–15932. doi: 10.1073/pnas.0407043101, PMID: 15509649 PMC528769

[ref16] Cisneros J. Belton T. B. B. Shum G. C. C. Molakal C. G. G. Wong Y. C. C. (2022). Mitochondria-lysosome contact site dynamics and misregulation in neurodegenerative diseases. Trends Neurosci. 45, 312–322. doi: 10.1016/j.tins.2022.01.005, PMID: 35249745 PMC8930467

[ref17] Claiborne A. B. English R. A. Kahn J. P. (2016). ETHICS OF NEW TECHNOLOGIES. Finding an ethical path forward for mitochondrial replacement. Science 351, 668–670. doi: 10.1126/science.aaf3091, PMID: 26842937

[ref18] Colaco A. Fernández-Suárez M. E. Shepherd D. Gal L. Bibi C. Chuartzman S.. (2020). Unbiased yeast screens identify cellular pathways affected in Niemann-pick disease type C. Life Sci. Allian. 3:e201800253. doi: 10.26508/lsa.201800253, PMID: 32487688 PMC7283134

[ref19] Elbaz-Alon Y. Rosenfeld-Gur E. Shinder V. Futerman A. H. Geiger T. Schuldiner M. (2014). A dynamic interface between vacuoles and mitochondria in yeast. Dev. Cell 30, 95–102. doi: 10.1016/j.devcel.2014.06.007, PMID: 25026036

[ref20] Fenton A. R. Peng R. Bond C. Hugelier S. Lakadamyali M. Chang Y. W.. (2024). FMRP regulates MFF translation to locally direct mitochondrial fission in neurons. Nat. Cell Biol. 26, 2061–2074. doi: 10.1038/s41556-024-01544-2, PMID: 39548330 PMC11628401

[ref21] Frosch M. Prinz M. (2024). Novel pathomechanistic insights into lysosomal storage disorders: how neuron-intrinsic cGAS-STING signaling drives disease progression. Signal Transduct. Tar. 9:203. doi: 10.1038/s41392-024-01901-5, PMID: 39147747 PMC11327262

[ref22] Garcia-Melero A. Reverter M. Hoque M. Meneses-Salas E. Koese M. Conway J. R.. (2016). Annexin A6 and late endosomal cholesterol modulate integrin recycling and cell migration. J. Biol. Chem. 291, 1320–1335. doi: 10.1074/jbc.M115.683557, PMID: 26578516 PMC4714218

[ref23] Giacomello M. Pyakurel A. Glytsou C. Scorrano L. (2020). The cell biology of mitochondrial membrane dynamics. Nat. Rev. Mol. Cell Biol. 21, 204–224. doi: 10.1038/s41580-020-0210-7 32071438

[ref24] Giamogante F. Barazzuol L. Maiorca F. Poggio E. Esposito A. Masato A.. (2024). A SPLICS reporter reveals α-synuclein regulation of lysosome-mitochondria contacts which affects TFEB nuclear translocation. Nat. Commun. 15:1516. doi: 10.1038/s41467-024-46007-2, PMID: 38374070 PMC10876553

[ref25] Gonzalez-Sanchez P. Satrustegui J. Palau F. Del Arco A. (2019). Calcium deregulation and mitochondrial bioenergetics in GDAP1-related CMT disease. Int. J. Mol. Sci. 20:403. doi: 10.3390/ijms20020403, PMID: 30669311 PMC6359725

[ref26] Haidar M. Timmerman V. (2017). Autophagy as an emerging common pathomechanism in inherited peripheral neuropathies. Front. Mol. Neurosci. 10:143. doi: 10.3389/fnmol.2017.00143, PMID: 28553203 PMC5425483

[ref27] Han Y. B. Li M. H. Qiu F. W. Zhang M. Zhang Y. H. (2017). Cell-permeable organic fluorescent probes for live-cell long-term super-resolution imaging reveal lysosome-mitochondrion interactions. Nat. Commun. 8:1307. doi: 10.1038/s41467-017-01503-6, PMID: 29101340 PMC5670236

[ref28] Harrington J. S. Ryter S. W. Plataki M. Price D. R. Choi A. M. K. (2023). Mitochondria in health, disease, and aging. Physiol. Rev. 103, 2349–2422. doi: 10.1152/physrev.00058.2021, PMID: 37021870 PMC10393386

[ref29] Höglinger D. Burgoyne T. Sanchez-Heras E. Hartwig P. Colaco A. Newton J.. (2019). NPC1 regulates ER contacts with endocytic organelles to mediate cholesterol egress. Nat. Commun. 10:4276. doi: 10.1038/s41467-019-12152-2, PMID: 31537798 PMC6753064

[ref30] Houlden H. King R. H. M. Muddle J. R. Warner T. T. Reilly M. M. Orrell R. W.. (2004). A novel RAB7 mutation associated with ulcero-mutilating neuropathy. Ann. Neurol. 56, 586–590. doi: 10.1002/ana.20281, PMID: 15455439

[ref31] Hutagalung A. H. Novick P. J. (2011). Role of Rab GTPases in membrane traffic and cell physiology. Physiol. Rev. 91, 119–149. doi: 10.1152/physrev.00059.2009, PMID: 21248164 PMC3710122

[ref32] Infante R. E. Wang M. L. Radhakrishnan A. Kwon H. J. Brown M. S. Goldstein J. L. (2008). NPC2 facilitates bidirectional transfer of cholesterol between NPC1 and lipid bilayers, a step in cholesterol egress from lysosomes. Proc. Natl. Acad. Sci. U. S. A. 105, 15287–15292. doi: 10.1073/pnas.0807328105, PMID: 18772377 PMC2563079

[ref33] Jordens I. Fernandez-Borja M. Marsman M. Dusseljee S. Janssen L. Calafat J.. (2001). The Rab7 effector protein RILP controls lysosomal transport by inducing the recruitment of dynein-dynactin motors. Curr. Biol. 11, 1680–1685. doi: 10.1016/S0960-9822(01)00531-0, PMID: 11696325

[ref34] Juhl A. D. Heegaard C. W. Werner S. Schneider G. Krishnan K. Covey D. F.. (2021). Quantitative imaging of membrane contact sites for sterol transfer between endo-lysosomes and mitochondria in living cells. Sci. Rep. 11:8927. doi: 10.1038/s41598-021-87876-7, PMID: 33903617 PMC8076251

[ref35] Kennedy B. E. Charman M. Karten B. (2012). Niemann-pick type C2 protein contributes to the transport of endosomal cholesterol to mitochondria without interacting with NPC1. J. Lipid Res. 53, 2632–2642. doi: 10.1194/jlr.M029942, PMID: 22962690 PMC3494252

[ref36] Khalil S. Holy M. Grado S. Fleming R. Kurita R. Nakamura Y.. (2017). A specialized pathway for erythroid iron delivery through lysosomal trafficking of transferrin receptor 2. Blood Adv. 1, 1181–1194. doi: 10.1182/bloodadvances.2016003772, PMID: 29296759 PMC5728310

[ref37] Kim J. E. Park S. Y. Kwak C. Lee Y. J. Song D. G. Jung J. W.. (2024). Glucose-mediated mitochondrial reprogramming by cholesterol export at TM4SF5-enriched mitochondria-lysosome contact sites. Cancer Commun. 44, 47–75. doi: 10.1002/cac2.12510, PMID: 38133457 PMC10794009

[ref38] Kim S. Wong Y. C. Gao F. Krainc D. (2021). Dysregulation of mitochondria-lysosome contacts by GBA1 dysfunction in dopaminergic neuronal models of Parkinson's disease. Nat. Commun. 12:1807. doi: 10.1038/s41467-021-22113-3, PMID: 33753743 PMC7985376

[ref39] Kleele T. Rey T. Winter J. Zaganelli S. Mahecic D. Lambert H. P.. (2021). Distinct fission signatures predict mitochondrial degradation or biogenesis. Nature 593, 435–439. doi: 10.1038/s41586-021-03510-6, PMID: 33953403

[ref40] Kostera-Pruszczyk A. Kosinska J. Pollak A. Stawinski P. Walczak A. Wasilewska K.. (2014). Exome sequencing reveals mutations in and in severe Charcot-Marie-Tooth disease. J. Peripher. Nerv. Syst. 19, 242–245. doi: 10.1111/jns.12088, PMID: 25403865

[ref41] Kyriakoudi S. Drousiotou A. Petrou P. P. (2021). When the balance tips: dysregulation of mitochondrial dynamics as a culprit in disease. Int. J. Mol. Sci. 22:4617. doi: 10.3390/ijms22094617, PMID: 33924849 PMC8124286

[ref42] Lackner L. L. (2019). The expanding and unexpected functions of mitochondria contact sites. Trends Cell Biol. 29, 580–590. doi: 10.1016/j.tcb.2019.02.009, PMID: 30929794 PMC6590070

[ref43] Lee J. W. (2015). Transmembrane 4 L six family member 5 (TM4SF5)-mediated epithelial-mesenchymal transition in liver diseases. Int. Rev. Cel Mol. Biol. 319, 141–163. doi: 10.1016/bs.ircmb.2015.06.004, PMID: 26404468

[ref44] Li J. Feng R. Yang W. Liang P. Qiu T. Zhang J.. (2024). Lysosomal iron accumulation and subsequent lysosomes-mitochondria iron transmission mediate PFOS-induced hepatocyte ferroptosis. Ecotoxicol. Environ. Saf. 284:116890. doi: 10.1016/j.ecoenv.2024.116890, PMID: 39146593

[ref45] Li L. Kim E. Yuan H. X. Inoki K. Goraksha-Hicks P. Schiesher R. L.. (2010). Regulation of mTORC1 by the Rab and Arf GTPases. J. Biol. Chem. 285, 19705–19709. doi: 10.1074/jbc.C110.102483, PMID: 20457610 PMC2888380

[ref46] Liesa M. Palacín M. Zorzano A. (2009). Mitochondrial dynamics in mammalian health and disease. Physiol. Rev. 89, 799–845. doi: 10.1152/physrev.00030.2008 19584314

[ref47] Lin J. X. Xu C. Y. Wu X. M. Che L. Li T. Y. Mo S. M.. (2023). Rab7a-mTORC1 signaling-mediated cholesterol trafficking from the lysosome to mitochondria ameliorates hepatic lipotoxicity induced by aflatoxin B1 exposure. Chemosphere 320:138071. doi: 10.1016/j.chemosphere.2023.138071, PMID: 36754296

[ref48] Markovinovic A. Greig J. Martín-Guerrero S. M. Salam S. Paillusson S. (2022). Endoplasmic reticulum-mitochondria signaling in neurons and neurodegenerative diseases. J. Cell Sci. 135:jcs248534. doi: 10.1242/jcs.248534, PMID: 35129196

[ref49] Martello A. Platt F. M. Eden E. R. (2020). Staying in touch with the endocytic network: the importance of contacts for cholesterol transport. Traffic 21, 354–363. doi: 10.1111/tra.12726, PMID: 32129938 PMC8650999

[ref50] Meneses-Salas E. Garcia-Melero A. Kanerva K. Blanco-Munoz P. Morales-Paytuvi F. Bonjoch J.. (2020). Annexin A6 modulates TBC1D15/Rab7/StARD3 axis to control endosomal cholesterol export in NPC1 cells. Cell. Mol. Life Sci. 77, 2839–2857. doi: 10.1007/s00018-019-03330-y, PMID: 31664461 PMC7326902

[ref51] Nguyen M. Wong Y. C. Ysselstein D. Severino A. Krainc D. (2019). Synaptic, mitochondrial, and lysosomal dysfunction in Parkinson's disease. Trends Neurosci. 42, 140–149. doi: 10.1016/j.tins.2018.11.001, PMID: 30509690 PMC6452863

[ref52] Onoue K. Jofuku A. Ban-Ishihara R. Ishihara T. Maeda M. Koshiba T.. (2013). Fis1 acts as a mitochondrial recruitment factor for TBC1D15 that is involved in regulation of mitochondrial morphology. J. Cell Sci. 126, 176–185. doi: 10.1242/jcs.111211, PMID: 23077178

[ref53] Otera H. Wang C. X. Cleland M. M. Setoguchi K. Yokota S. Youle R. J.. (2010). Mff is an essential factor for mitochondrial recruitment of Drp1 during mitochondrial fission in mammalian cells. J. Cell Biol. 191, 1141–1158. doi: 10.1083/jcb.201007152, PMID: 21149567 PMC3002033

[ref54] Pankiv S. Alemu E. A. Brech A. Bruun J. A. Lamark T. Overvatn A.. (2010). FYCO1 is a Rab7 effector that binds to LC3 and PI3P to mediate microtubule plus end-directed vesicle transport. J. Cell Biol. 188, 253–269. doi: 10.1083/jcb.200907015 20100911 PMC2812517

[ref55] Pedersen S. F. Flinck M. Pardo L. A. (2021). The interplay between dysregulated ion transport and mitochondrial architecture as a dangerous liaison in Cancer. Int. J. Mol. Sci. 22:5209. doi: 10.3390/ijms22105209, PMID: 34069047 PMC8156689

[ref56] Peng W. Schroder L. F. Song P. Wong Y. C. Krainc D. (2023). Parkin regulates amino acid homeostasis at mitochondria-lysosome (M/L) contact sites in Parkinson's disease. Sci. Adv. 9:eadh3347. doi: 10.1126/sciadv.adh3347, PMID: 37467322 PMC10355824

[ref57] Peng W. Wong Y. C. Krainc D. (2020). Mitochondria-lysosome contacts regulate mitochondrial Ca2+ dynamics via lysosomal TRPML1. Proc. Natl. Acad. Sci. U. S. A. 117, 19266–19275. doi: 10.1073/pnas.2003236117, PMID: 32703809 PMC7430993

[ref58] Peralta E. R. Martin B. C. Edinger A. L. (2010). Differential effects of TBC1D15 and mammalian Vps39 on Rab7 activation state, lysosomal morphology, and growth factor dependence. J. Biol. Chem. 285, 16814–16821. doi: 10.1074/jbc.M110.111633, PMID: 20363736 PMC2878074

[ref59] Perera R. M. Zoncu R. (2016). The lysosome as a regulatory hub. Annu. Rev. Cell Dev. Biol. 32, 223–253. doi: 10.1146/annurev-cellbio-111315-125125, PMID: 27501449 PMC9345128

[ref60] Petkovic M. O'Brien C. E. Jan Y. N. (2021). Interorganelle communication, aging, and neurodegeneration. Genes Dev. 35, 449–469. doi: 10.1101/gad.346759.120, PMID: 33861720 PMC8015714

[ref61] Phillips M. J. Voeltz G. K. (2016). Structure and function of ER membrane contact sites with other organelles. Nat. Rev. Mol. Cell Biol. 17, 69–82. doi: 10.1038/nrm.2015.8, PMID: 26627931 PMC5117888

[ref62] Pijuan J. Cantarero L. Benito D. N. D. Altimir A. Altisent-Huguet A. Diaz-Osorio Y.. (2022). Mitochondrial dynamics and mitochondria-lysosome contacts in neurogenetic diseases. Front. Neurosci. 16:784880. doi: 10.3389/fnins.2022.784880, PMID: 35177962 PMC8844575

[ref63] Qi J. S. Li Q. Q. Xin T. L. Lu Q. X. Lin J. Y. Zhang Y.. (2024). MCOLN1/TRPML1 in the lysosome: a promising target for autophagy modulation in diverse diseases. Autophagy 20, 1712–1722. doi: 10.1080/15548627.2024.2333715, PMID: 38522082 PMC11262240

[ref64] Qiu K. Q. Zou W. W. Fang H. B. Hao M. G. Mehta K. Tian Z. Q.. (2022). Light-activated mitochondrial fission through optogenetic control of mitochondria-lysosome contacts. Nat. Commun. 13:4303. doi: 10.1038/s41467-022-31970-5, PMID: 35879298 PMC9314359

[ref65] Rosencrans W. M. Rajendran M. Bezrukov S. M. Rostovtseva T. K. (2021). VDAC regulation of mitochondrial calcium flux: from channel biophysics to disease. Cell Calcium 94:102356. doi: 10.1016/j.ceca.2021.102356, PMID: 33529977 PMC7914209

[ref66] Su C. Zheng C. (2021). When Rab GTPases meet innate immune signaling pathways. Cytokine Growth Factor Rev. 59, 95–100. doi: 10.1016/j.cytogfr.2021.01.002, PMID: 33608190

[ref67] Sun S. Yu W. Xu H. Li C. Zou R. Wu N. N.. (2022). TBC1D15-Drp1 interaction-mediated mitochondrial homeostasis confers cardioprotection against myocardial ischemia/reperfusion injury. Metabolism 134:155239. doi: 10.1016/j.metabol.2022.155239, PMID: 35680100

[ref68] Todkar K. Ilamathi H. S. Germain M. (2017). Mitochondria and lysosomes: discovering bonds. Front. Cell Dev. Biol. 5:106. doi: 10.3389/fcell.2017.00106, PMID: 29270406 PMC5725469

[ref69] Trivedi P. C. Bartlett J. J. Pulinilkunnil T. (2020). Lysosomal biology and function: modern view of cellular debris bin. Cells 9:1131. doi: 10.3390/cells9051131, PMID: 32375321 PMC7290337

[ref70] Valm A. M. Cohen S. Legant W. R. Melunis J. Hershberg U. Wait E.. (2017). Applying systems-level spectral imaging and analysis to reveal the organelle interactome. Nature 546, 162–167. doi: 10.1038/nature22369, PMID: 28538724 PMC5536967

[ref71] Volodarsky M. Kerkhof J. Stuart A. Levy M. Brady L. I. Tarnopolsky M.. (2021). Comprehensive genetic sequence and copy number analysis for Charcot-Marie-Tooth disease in a Canadian cohort of 2517 patients. J. Med. Genet. 58, 284–288. doi: 10.1136/jmedgenet-2019-106641, PMID: 32376792

[ref72] Wang K. Ho C. C. Li X. Y. Hou J. F. Luo Q. P. Wu J. H.. (2024). Matrix stiffness regulates mitochondria-lysosome contacts to modulate the mitochondrial network, alleviate the senescence of MSCs. Cell Proliferat:e13746. doi: 10.1111/cpr.13746, PMID: 39353686 PMC11839199

[ref73] Wang H. Qiao Z. Luan K. Xiang W. Chang X. Zhang Y.. (2024). Identification of a new retigabine derivative with improved photostability for selective activation of neuronal Kv7 channels and antiseizure activity. Epilepsia 65, 2923–2934. doi: 10.1111/epi.18092 39140981

[ref74] Ward D. M. Cloonan S. M. (2019). Mitochondrial Iron in human health and disease. Annu. Rev. Physiol. 81, 453–482. doi: 10.1146/annurev-physiol-020518-114742, PMID: 30485761 PMC6641538

[ref75] Wolf C. Pouya A. Bitar S. Pfeiffer A. Bueno D. Arndt S.. (2022). GDAP1 loss of function inhibits the mitochondrial pyruvate dehydrogenase complex by altering the actin cytoskeleton. Commun. Biol. 5:541. doi: 10.1038/s42003-022-03487-6, PMID: 35662277 PMC9166793

[ref76] Wong Y. C. Jayaraj N. D. Belton T. B. Shum G. C. Ball H. E. Ren D.. (2023). Misregulation of mitochondria-lysosome contact dynamics in Charcot-Marie-tooth type 2B disease Rab7 mutant sensory peripheral neurons. Proc. Natl. Acad. Sci. U. S. A. 120:e2313010120. doi: 10.1073/pnas.2313010120, PMID: 37878717 PMC10622892

[ref77] Wong Y. C. Kim S. Cisneros J. Molakal C. G. Song P. P. Lubbe S. J.. (2022). Mid51/Fis1 mitochondrial oligomerization complex drives lysosomal untethering and network dynamics. J. Cell Biol. 221:e202206140. doi: 10.1083/jcb.202206140, PMID: 36044022 PMC9437119

[ref78] Wong Y. C. Kim S. Peng W. Krainc D. (2019a). Regulation and function of mitochondria-lysosome membrane contact sites in cellular homeostasis. Trends Cell Biol. 29, 500–513. doi: 10.1016/j.tcb.2019.02.004, PMID: 30898429 PMC8475646

[ref79] Wong Y. C. Peng W. Krainc D. (2019b). Lysosomal regulation of inter-mitochondrial contact fate and motility in Charcot-Marie-tooth type 2. Dev. Cell 50, 339–354.e4. doi: 10.1016/j.devcel.2019.05.033, PMID: 31231042 PMC6726396

[ref80] Wong Y. C. Ysselstein D. Krainc D. (2018). Mitochondria-lysosome contacts regulate mitochondrial fission via RAB7 GTP hydrolysis. Nature 554, 382–386. doi: 10.1038/nature25486, PMID: 29364868 PMC6209448

[ref81] Wos M. Szczepanowska J. Pikula S. Tylki-Szymanska A. Zablocki K. Bandorowicz-Pikula J. (2016). Mitochondrial dysfunction in fibroblasts derived from patients with Niemann-pick type C disease. Arch. Biochem. Biophys. 593, 50–59. doi: 10.1016/j.abb.2016.02.012, PMID: 26869201

[ref82] Wu H. X. Carvalho P. Voeltz G. K. (2018). Here, there, and everywhere: the importance of ER membrane contact sites. Science 361:eaan5835. doi: 10.1126/science.aan5835, PMID: 30072511 PMC6568312

[ref83] Xie Y. Zhang W. Peng T. Wang X. Lian X. He J.. (2024). TBC1D15-regulated mitochondria-lysosome membrane contact exerts neuroprotective effects by alleviating mitochondrial calcium overload in seizure. Sci. Rep. 14:23782. doi: 10.1038/s41598-024-74388-3, PMID: 39390030 PMC11467349

[ref84] Xu P. Swain S. Novorolsky R. J. Garcia E. Huang Z. Snutch T. P.. (2024). The mitochondrial calcium uniporter inhibitor Ru265 increases neuronal excitability and reduces neurotransmission via off-target effects. Br. J. Pharmacol. 181, 3503–3526. doi: 10.1111/bph.16425, PMID: 38779706 PMC11309911

[ref85] Ye H. Robak L. A. Yu M. Cykowski M. Shulman J. M. (2023). Genetics and pathogenesis of Parkinson’s syndrome. Annu. Rev. Pathol. 18, 95–121. doi: 10.1146/annurev-pathmechdis-031521-034145, PMID: 36100231 PMC10290758

[ref86] Young P. De Jonghe P. Stogbauer F. Butterfass-Bahloul T. (2008). Treatment for Charcot-Marie-Tooth disease. Cochrane Database Syst. Rev. 2008:CD006052. doi: 10.1002/14651858.CD006052.pub2 18254090 PMC6718225

[ref87] Yu W. J. Sun S. Q. Xu H. X. Li C. Y. Ren J. Zhang Y. M. (2020). TBC1D15/RAB7-regulated mitochondria-lysosome interaction confers cardioprotection against acute myocardial infarction-induced cardiac injury. Theranostics 10, 11244–11263. doi: 10.7150/thno.46883, PMID: 33042281 PMC7532681

[ref88] Zhang M. Liu P. Dwyer N. K. Christenson L. K. Fujimoto T. Martinez F.. (2002). MLN64 mediates mobilization of lysosomal cholesterol to steroidogenic mitochondria. J. Biol. Chem. 277, 33300–33310. doi: 10.1074/jbc.M200003200, PMID: 12070139

[ref89] Zhang X. M. Walsh B. Mitchell C. A. Rowe T. (2005). TBC domain family, member 15 is a novel mammalian Rab GTPase-activating protein with substrate preference for Rab7. Biochem. Bioph Res. Co. 335, 154–161. doi: 10.1016/j.bbrc.2005.07.070, PMID: 16055087

[ref90] Zhu M. Wang X. Q. (2020). Regulation of mTORC1 by small GTPases in response to nutrients. J. Nutr. 150, 1004–1011. doi: 10.1093/jn/nxz301, PMID: 31965176

[ref91] Züchner S. Mersiyanova I. V. Muglia M. Bissar-Tadmouri N. Rochelle J. Dadali E. L.. (2004). Mutations in the mitochondrial GTPase mitofusin 2 cause Charcot-Marie-tooth neuropathy type 2A. Nat. Genet. 36:660. doi: 10.1038/ng0604-660 15064763

